# Comparison of Different Weight Scalars to Dose Sugammadex for the Reversal of Neuromuscular Blockade in Morbidly Obese Patients: A Systematic Review

**DOI:** 10.7759/cureus.57057

**Published:** 2024-03-27

**Authors:** Yamini Subramani, Manikandan Rajarathinam, Anita Dabirzadeh, Qutaiba Tawfic, Sarah Krause, Yasin Avci, Mahesh Nagappa

**Affiliations:** 1 Anesthesiology and Perioperative Medicine, London Health Sciences Centre, Western University, London, Canada; 2 Anesthesiology and Perioperative Medicine, London Health Sciences Centre, Western University, London, CAN; 3 Medical Sciences, Western University, London, CAN; 4 Interdisciplinary Arts and Science, Western University, London, CAN

**Keywords:** reversal, neuromuscular blockade, sugammadex, overweight, obesity

## Abstract

This systematic review was conducted to evaluate the optimal weight scalar to dose sugammadex in a morbidly obese (MO) patient population (BMI≥40 kg/m^2^). The primary outcome was recovery time from moderate neuromuscular blockade (NMB) or deep NMB. Secondary outcomes included time to extubation and incidence of postoperative residual curarization (PORC). Eight randomized controlled trials (RCTs) involving 645 participants were included. The different dose scalars included were total body weight (TBW), ideal body weight (IBW), 20% corrected body weight (CBW) and 40% CBW). A dose of 2 mg/kg of sugammadex based on 40% CBW and a 4 mg/kg dose of sugammadex based on 40% CBW provide a reliable and timely reversal of moderate and deep NMB respectively in the MO patients.

## Introduction and background

Neuromuscular blockade (NMB) is essential to facilitate intubation and create ideal operating conditions for bariatric surgical procedures. Sugammadex has been developed as a unique reversal agent for amino steroid drugs-induced NMB, particularly for rocuronium. Its mechanism involves encapsulating and inactivating rocuronium, forming tight 1:1 complexes [1]. Morbidly obese (MO) patients (BMI≥40 kg/m^2^) are prone to critical respiratory events such as the inability to maintain a patent airway, hypoventilation and residual NMB [2]. This population exhibits a higher incidence of residual curarization vs. non-obese patients. Sugammadex has been proven to be a safe and effective alternative to neostigmine in reversing NMB in MO patients, resulting in a shorter recovery time to a Train of Four (TOF) of 0.9 [2-5]. The optimal sugammadex dosing prevents postoperative residual curarization (PORC) in this patient population. Eleveld et al. first described PORC, attributing it to an inadequate sugammadex dose relative to the block degree [6]. PORC and recurarization might increase the incidence of postoperative pulmonary complications (PPCs), contributing significantly to surgical morbidity and mortality [7,8]. The recommended dose of sugammadex for immediate (within 3-5 minutes), deep (1-2 post-tetanic counts (PTCs)) and moderate reversal (at the appearance of second twitch (T2)) rocuronium-induced NMB are 16, 4 and 2 mg/kg, respectively [1]. 

While sugammadex can be administered based on actual body weight for normal-weight patients with similar total body weight (TBW), lean body weight (LBW) and ideal body weight (IBW), the MO patients necessitate a distinct dosing regimen due to certain physiological changes [9]. Considering the high cost of sugammadex, it is imperative to establish evidence for appropriately dosing sugammadex in MO patients, avoiding unnecessary overdosing and reducing the associated economic burden [7]. However, given the risk of PORC as described above, underdosing sugammadex is considered more dangerous than overdosing, emphasizing the need for an optimal weight scalar for sugammadex dosing. The literature presents conflicting views on the ideal weight-based dosing scalar for sugammadex in MO patients. This study aims to conduct a comprehensive and systematic literature review to determine the optimal weight scalar for sugammadex dosing in the MO patient population.

## Review

Methods

This systematic review adhered to the Cochrane systematic review guidelines and followed the reporting guidelines outlined in the Preferred Reporting Items for Systematic Reviews and Meta-Analyses (PRISMA). 

Study selection criteria

A systematic search for randomized controlled trials (RCTs) comparing different weight-based scalars for dosing sugammadex to reverse moderate NMB (TOF 2) in MO patients with a BMI of 40 kg/m^2^ was conducted. For inclusion, trials had to report on the primary endpoint: recovery times to a TOF ratio of 0.9 after sugammadex administration. Secondary outcomes such as extubation time and the incidence of PORC were also considered in some trials. 

Literature search

The following databases were searched for relevant studies in the English language, which were performed on the following databases: PubMed, Medline, Embase, Cochrane Central Register of Controlled Trials, Web of Science, Scopus, and CINAHL. The search covered the period from 1946 to July 2020. The reference lists of the retrieved studies were reviewed to identify any relevant articles. Keywords used in the search were obesity, overweight, sugammadex, selective relaxant binding agent (SRBA), and various terms related to obesity. Retrospective trials and case reports were excluded. Two independent reviewers (Y.S. and M.N.) screened the citations and retrieved the full text of potentially eligible articles. Two authors (Y.S. and M.N.) independently evaluated the methodological quality of the articles that met the inclusion criteria, utilizing the Cochrane risk of bias tool. 

Data extraction

Two reviewers (Y.S. and M.N.) independently extracted data using standardized forms. The extracted information encompassed the following elements: (i) study details, (ii) dose of sugammadex used, (iii) recovery time to TOF of 0.9, (iv) incidence of PORC, and (v) extubation time. 

Outcomes

The primary outcome was time to recovery from moderate NMB (from the emergence of T2 to TOF ratio > 0.9) or deep NMB (from the emergence of PTC 1-5 to TOF ratio > 0.9) with sugammadex vs. neostigmine groups. Secondary outcomes were the time to extubation and the incidence of PORC.

Results

**Figure 1:**
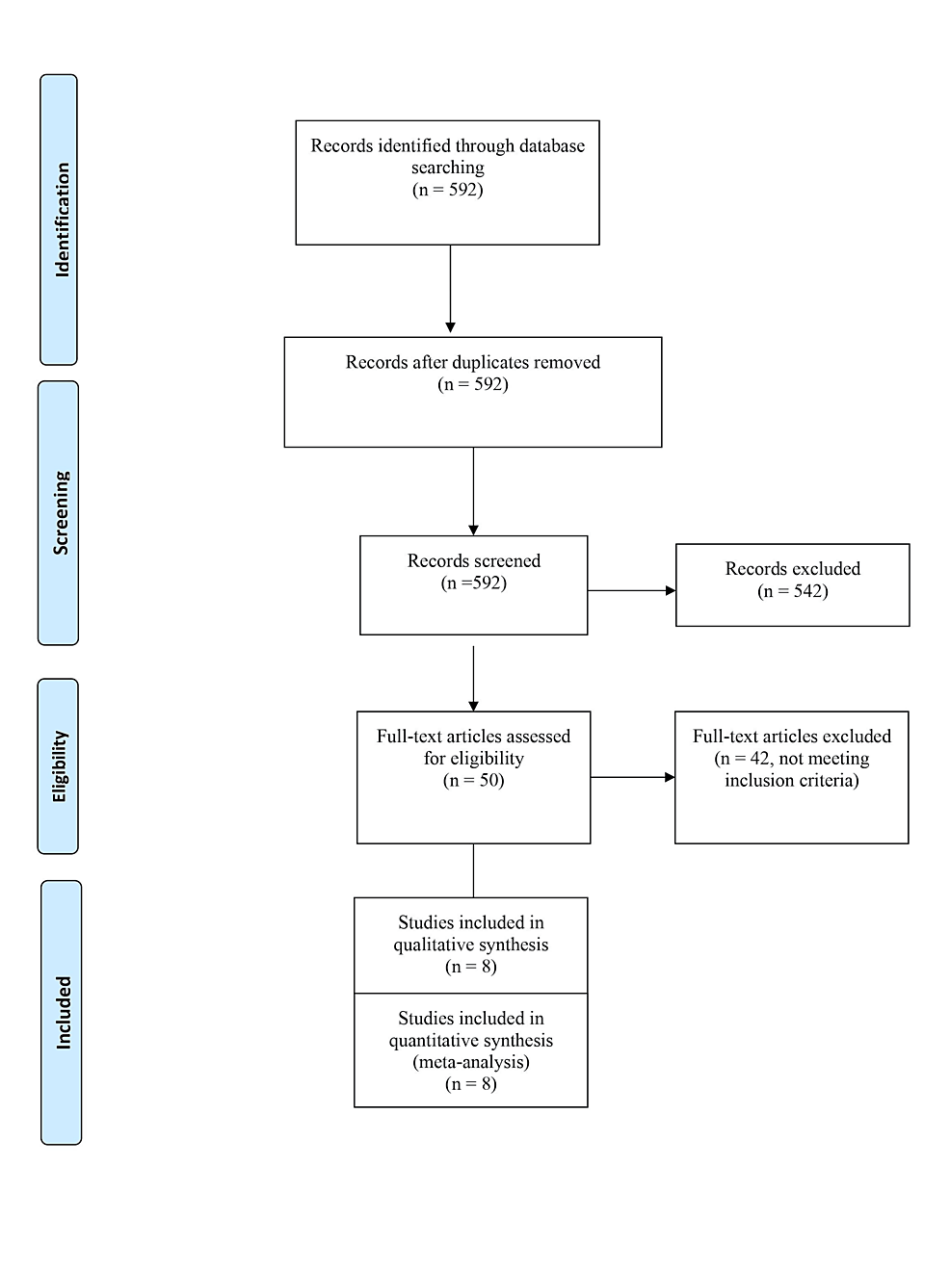
PRISMA flow diagram PRISMA: Prferred Reporting Items for Systematic Reviews and Meta-Analyses

A total of 592 studies were identified from the initial search and further screened by title and abstract, resulting in the selection of 50 studies for full-text review. 8 RCTs involving 645 participants were ultimately included in the analysis (Figure 1) [10-17].

All the included trials were published in English. The patients in these trials underwent various bariatric surgical procedures under general anesthesia, with rocuronium as an NMB agent. Different doses of sugammadex, based on various weight scalars, were administered as the reversal drug. A summary of the systematic review of the included studies is provided in Table 1. 

**Table 1:** Systematic review C1, C2, C3, C4: Comparison of sugammadex dosage scalars; IBW: Ideal body weight; CBW: Corrected body weight; TBW: Total body weight; TOF: Train of Four; PORC: Postoperative residual curarization; s: Second; Extubation time: Time from administration of sugammadex to extubation

Author	C1	C2	C3	C4	Number of patients C1/C2/ C3/C4	Intensity of block at reversal	Time to TOF>0.9 for CI (s): Mean (SD)	Time to TOF>0.9 for C2 (s): Mean (SD)	Time to TOF>0.9 for C3 (s): Mean (SD)	Time to TOF>0.9 for C4 (s): Mean (SD)	PORC: C1	PORC: C2	PORC: C3	PORC: C3	Extubation time for C1 (s): Mean (SD)	Extubation time for C2 (s): Mean (SD)	Extubation time for C3 (s): Mean (SD)	Extubation time for C4 (s): Mean (SD)
Sanfilippo 2013 [10]	2 mg/kg IBW	2 mg/kg TBW	NA/-	NA/-	20/20	Moderate	151 (44)	121 (55)	NA/-	NA/-	0	0	NA/-	NA/-	NA/-	NA/-	NA/-	NA/-
Loupec 2016 [11]	4 mg/kg IBW	2 mg/kg IBW	1 mg/kg IBW	NA/-	15/17/18	Deep	255 (63)	429 (102)	581 (154)	NA/-	1	4	14	NA/-	22.3 (5.15)	24.6 (10.99)	20.16 (5.95)	NA/-
Van Lancker 2011 [12]	2 mg/kg IBW	2 mg/kg CBW (20%)	2 mg/kg CBW (40%)	2 mg/kg TBW	25/25/25/28	Moderate	188.9 (84.4)	154.6 (59.7)	112.5 (30.3)	128.8 (47)	0	0	0	0	318.4 (122) s	306.9 (184.7) s	255.1 (119.9) s	326.8 (107.9)s
Duarte et al 2018 [13]	2 mg/kg IBW	2 mg/kg CBW (20%)	2 mg/kg CBW (40%)	NA/-	20/19/17	Moderate	225.2 (81.2)	173.9 (86.8)	174.1 (74.9)	NA/-	0	0	0	NA/-	NA/-	NA/-	NA/-	NA/-
Ornek et al 2020 [14]	2 mg/kg IBW	2 mg/kg CBW (40%)	2 mg/kg TBW	NA/-	20/20/20	Moderate	202.65 (79.9)	170.45 (146.16)	137.05 (106.10)	NA/-	0	0	0	NA/-	NA/-	NA/-	NA/-	NA/-
El-Rahman et al 2017 [15]	1.5 mg/kg IBW	2 mg/kg IBW	4 mg/kg IBW	NA/-	60/60/60	Moderate	150 (18)	150 (18)	138 (18)	NA/-	0	0	0	NA/-	288 (42)	288 (42)	288 (42)	NA/-
Elfawy et al 2018 [16]	2 mg/kg IBW	2 mg/kg CBW (40%)	2 mg/kg TBW	NA/-	20/20/20	Moderate	176.3 (5.44)	141.85 (5.184)	137.9 (3.307)	NA/-	NA/-	NA/-	NA/-	NA/-	293.05 (13.008)	299.3 (8.033)	297.05 (6.26)	NA/-
Deming Li et al 2021 [17]	4 mg/kg CBW (40%)	4 mg/kg TBW	NA/-	NA/-	49/47	Deep	144 (39)	125 (43.2)	NA/-	NA/-	0	0	NA/-	NA/-	NA/-	NA/-	NA/-	NA/-

**Figure 2:**
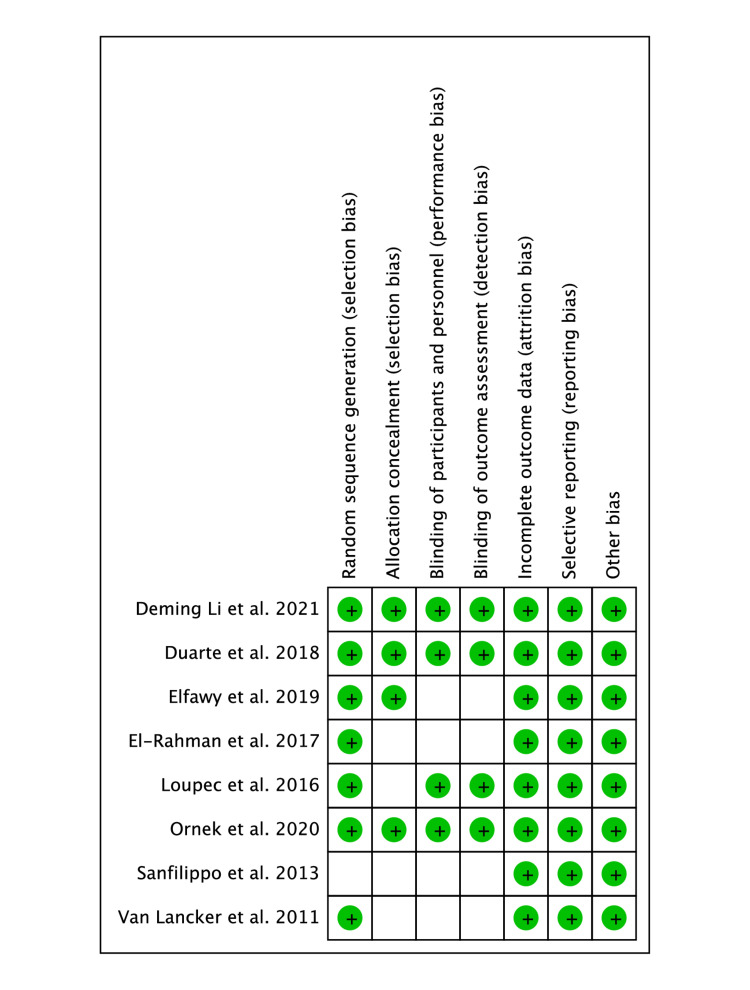
Risk of bias summary [10-17] Green circles: Low risk of bias; Unchecked boxes: Unclear risk of bias

The quality assessment of the included studies was done using the Cochrane risk of bias tool, which revealed that all studies had some degree of risk of bias in at least one domain (Figure 2). 

The different dose scalars used in the included studies were TBW, IBW, 20% CBW and 40% CBW. The CBW is calculated using the following formulae:

40% of CBW (40%CBW) = IBW + 0.4 (TBW − IBW)

20% of CBW (20%CBW) = IBW + 0.2 (TBW − IBW

Due to the heterogeneity in the doses of sugammadex across the included studies, a meta-analysis was not performed. 

Sanfilippo et al. examined the safety and efficacy of sugammadex doses based on IBW [10]. In their study, 40 patients were randomized to receive 2 mg/kg of sugammadex to reverse moderate NMB based on IBW or TBW. The recovery times to a TOF ratio of 0.9 were similar in both groups (P = 0.07), and no PORC was observed (Table 1). Loupec et al. conducted an RCT involving patients with deep NMB [11]. The patients were assigned to receive sugammadex at 4 mg/kg, 2 mg/kg, and 1 mg/kg of IBW. The results indicated a significantly shorter mean recovery time from deep NMB in the 4 mg/kg group (n = 14; mean (SD): 255 (63) s) vs. 2 mg/kg group (n = 13; mean (SD): 429 (102) s) or 1 mg/kg group (n = 4; mean (SD): 581 (154) s). A TOF≥0.9 within 10 minutes after sugammadex administration, which is a marker of successful reversal from NMB, was achieved in 93%, 77% and 22% of patients in 4 mg/kg, 2 mg/kg and 1 mg/kg groups, respectively (p < 0.05) (Table 1).

Van Lancker et al. conducted an RCT involving 100 MO patients who were allocated to receive 2 mg/kg of sugammadex based on various weight scalars: IBW, IBW + 20%, IBW + 40%, and TBW [12]. No residual paralysis was observed in any patient. The study revealed a significant difference among the four groups in terms of recovery time from NMB (p < 0.0001), with the IBW + 40% group demonstrating the fastest recovery (mean (SD): 112.5 (30.3) s). However, there was no significant difference in extubation time among the four groups (p = 0.253) (Table 1).

Duarte et al. conducted an RCT with 56 patients, dividing them into groups receiving 2 mg/kg of sugammadex based on IBW, 20% and 40% of CBW to reverse a moderate NMB [13]. The study found no significant difference between the three groups regarding recovery time from NMB (p = 0.088) (Table 1). Ornek et al. randomized 60 patients to compare the 2 mg/kg doses of sugammadex based on IBW, 40% CBW and TBW to reverse moderate NMB [14]. The results indicated that the time to TOF ratio of 0.9 and time to extubation was the shortest in the TBW group, while these times were the longest in the IBW group (p = 0.05, 0.018, respectively) (Table 1). El-Rahman et al. randomized 180 MO patients into three groups based on sugammadex dose of 1.5 mg/kg, 2 mg/kg, and 4 mg/kg administrated according to IBW [15]. The time to reversal was significantly longer in 1.5 mg/kg and 2 mg/kg vs. 4 mg/kg IBW of sugammadex (p = 0.000, 0.005, respectively). However, the difference between the 1.5 and 2 mg/kg groups was insignificant. The extubation time showed no significant difference among the three groups (P>0.05) (Table 1).

Elfawy et al. randomized 60 patients to compare 2 mg/kg doses of sugammadex based on IBW, 40% CBW, and TBW to reverse moderate NMB [16]. The mean reversal time decreased from 176.30±5.44 seconds in the IBW group to 141.85±5.184 seconds in the 40% CBW group and decreased to 137.9±3.307 seconds in the TBW group. Pairwise comparisons revealed statistically significant decreases in reversal time from the IBW to the 40% CBW group (p<0.001) and from the IBW to the TBW group (p<0.001). However, the decrease in reversal time from the 40% CBW group to the TBW group was not statistically significant (p = 0.062). The extubation time was insignificantly different in the three groups (P = 0.120) (Table 1).

Deming Li et al. randomized patients with deep NMB to receive sugammadex at 4 mg/kg based on 40% CBW and TBW [17]. The mean (SD) recovery times from the start of sugammadex administration to a TOF ratio of 0.9 were 2.2 (0.7) and 2.0 (0.7) minutes in the CBW and TBW groups, respectively. The study concluded that 40% of CBW is non-inferior to TBW in reversing deep NMB in MO patients (Table 1).

Discussion

Our review identifies 40% CBW as the optimal dose scalar for sugammadex in MO patients. Although the manufacturer recommends titrating the sugammadex dose according to the TBW, given its high hydrophilicity and distribution primarily into the plasma within the central compartment, it is advisable to consider dosing based on IBW or CBW in MO patients [16,18,19]. IBW represents the weight believed to be optimal for health, and the method devised by Broca is commonly used to calculate IBW for men and women [19]. El-Rahman et al. suggested the effective administration of sugammadex based on IBW in MO patients, evaluating three dosages (1.5, 2, and 4 mg/kg IBW) [15]. Although the TOF recovery time was significantly shorter with the 4 mg/kg IBW, the differences between groups were deemed limited clinical importance. Recovery times to a TOF of 0.9 ranged between two and three minutes in all three groups. A trend was observed, indicating a higher need for a second sugammadex rescue dose in the groups receiving 1.5 and 2 mg/kg IBW as the first dose, though not statistically significant. Other dose-finding studies cited by the authors indicated that a dose lower than 2 mg/kg of sugammadex could effectively reverse a moderate NMB induced by rocuronium, but these studies included non-obese patients [20,21]. This finding, supported by similar studies, contrasts with others that found that IBW alone was insufficient to reverse moderate NMB [10,11,13]. Llaurado et al.’s prospective observational study reported that an IBW-based dose successfully reversed paralysis in only 77% of patients, with 23% requiring a second dose of sugammadex due to the absence of recovery of T4/T1>0.90 within 3 minutes [22]. However, it’s important to note that the time used to assess the success or failure of the reversal process was only three minutes for deep and two minutes for moderate NMB in this study. Additionally, the total dose of rocuronium and the duration of the surgery were higher in Llaurado et al.’s study than in the study by El-Rahman et al.'s study [15,22]. Duarte et al. reinforce these findings from a biochemical and pharmacological perspective, asserting that rocuronium and sugammadex are hydrophilic compounds with no affinity for fatty tissue [13]. If rocuronium can be administered based on IBW, the same principle holds with sugammadex, as it inactivates rocuronium at a molecular level in a 1:1 proportion [23]. Similarly, Loupec et al.’s study proposes a dose of 4 mg/kg IBW for sugammadex as clinically effective and reasonable as 1 mg/kg and 2 mg/kg IBW doses resulted in residual NMB in a significant number of patients [11].

Several studies have supported the conventional TBW-based sugammadex dosing, as the manufacturer recommended. Carron et al. have suggested that dosing sugammadex based on TBW achieves a quick and reliable TOF ratio of 1 [24]. The study proposes that sugammadex maintains an acceptable safety profile, even at higher doses [1]. Further, an insufficient sugammadex dose may lead to recurarization after a high dose or due to altered metabolism and elimination of rocuronium [12,25]. Therefore, while it is reasonable to consider IBW-based dosing of sugammadex, given its pharmacokinetic profile, TBW-based dosing continues to be considered safe and effective for complete reversal from NMB in MO patients [24]. Ornek et al. found that increased doses of sugammadex resulted in more effective and faster recovery from NMB, comparing 2 mg/kg of sugammadex based on TBW, IBW and 40% CBW. They suggested that TBW-based sugammadex dosing might be more appropriate for a safe and effective reversal of moderate rocuronium-induced NMB in MO patients [14]. A recent study by Harrow et al. compared 2 mg/kg and 4 mg/kg doses of sugammadex based on IBW and TBW, revealing that IBW-based dosing did not offer any safety advantage, but led to delayed recovery times compared to dosing by TBW. The authors concluded that MO patients should receive sugammadex based on TBW, regardless of the block depth or muscle relaxant choice. Notably, this study, which is not included in our review, randomized patients with moderate and deep NMB to each study group, pooling the time to recovery to a TOF ratio ≥ 0.9 across the depth of the block [26]. The study by Ornek et al. identified certain slow responders, as reported in a dosing study on lean individuals with sugammadex [14,27]. They also emphasize that the recovery of the TOF ratio to 0.9 is insufficient to achieve optimal reversal and prevent upper airway obstruction in obese patients. They advocate for a TOF ratio 1.0, supporting TBW dosing based on the aforementioned arguments. 

CBW or adjusted body weight was initially employed to calculate caloric requirements in MO patients [28]. Drug administration based on CBW has recently been proposed in MO patients [29,30]. An observational study concluded that 4 mg/kg of IBW plus 35-50% can reverse deep NMB in MO patients [31]. In a similar study, Van Lancker et al. administered sugammadex based on 40% CBW, 20% CBW, IBW, and CBW. They observed longer recovery times in the IBW and 20% CBW groups than in 40% CBW and TBW-based sugammadex dosing, recommending a 40% CBW-based dose to reverse moderate NMB [12]. Although the authors did not notice a clinically significant difference in the reversal time between the IBW, CBW, and TBW groups, they proposed the 40% CBW dose as a safe dose to prevent recurarization. This recommendation was based on the study by Eleveld et al. [6] , which demonstrated that a lower dose of sugammadex is sufficient to form complexes with rocuronium molecules in the central compartment but inadequate to sustain the redistribution of rocuronium from the peripheral to the central compartment. Elfawy et al. compared 2 mg/kg of sugammadex based on TBW, IBW, and 40% CBW to reverse moderate NMB. The study showed a statistically significant difference in recovery time for doses based on IBW versus CBW and TBW. However, there was no difference in recovery time between CBW and TBW. Additionally, the cost of reversal with sugammadex in a 140 kg person based on CBW was $74.56 compared to $149.12 based on TBW, with comparable effects and outcomes [16]. In a recent RCT, Li et al. demonstrated that 4 mg/kg CBW of sugammadex is sufficient and safe for reversing deep NMB resulting from a continuous infusion of rocuronium in MO patients [17].

Limitations

This systematic review has certain limitations that should be considered. The quality of the included trials limits the overall reliability of the evidence in this systematic review. All trials had at least one domain with an unclear risk of bias, introducing the potential for both overestimation and underestimation of the true intervention effect. While all studies defined the characteristic of the NMB as moderate or deep at the time of reversal, the total dose of rocuronium and the time between the last dose and administration of sugammadex were not standardized across included trials. This inconsistency highlights the need for future standardized studies addressing these aspects to enhance the reliability and comparability of findings. 

## Conclusions

Drawing from the existing evidence, we conclude that a dose of 2 mg/kg of sugammadex based on 40% CBW and a dose of 4 mg/kg based on 40% CBW offers a reliable and timely reversal of moderate and deep NMB, respectively. This conclusion holds for MO patients undergoing laparoscopic bariatric surgery without introducing any risk of residual NMB cost-effectively. It is important to note that higher doses of sugammadex based on TBW are unnecessary in this specific population.
